# Supplementary material for: A high-resolution mRNA expression time course of embryonic development in zebrafish
Source: eLife. 2017 Nov 16;6:e30860. doi: 10.7554/eLife.30860 (PMC5690287; doi:10.7554/eLife.30860)
Supplement: Supplementary file 6. [file elife-30860-supp6.zip › biolayout-clusters-files/Cluster154-genes.html]

Cluster154


# Cluster154: Genes

| Ensembl ID | Gene Name | Chr | Start | End | Biotype |
| --- | --- | --- | --- | --- | --- |
| ENSDARG00000058445 | ENSDARG00000058445 | 1 | 8550817 | 8565832 | protein\_coding |
| ENSDARG00000101645 | ENSDARG00000101645 | 4 | 71140340 | 71166300 | protein\_coding |
| ENSDARG00000013050 | abcc12 | 7 | 37887350 | 37912073 | protein\_coding |
| ENSDARG00000014727 | acox1 | 6 | 18024741 | 18040346 | protein\_coding |
| ENSDARG00000036846 | anks4b | 3 | 60354303 | 60387724 | protein\_coding |
| ENSDARG00000057064 | enpep | 13 | 12411159 | 12448913 | protein\_coding |
| ENSDARG00000005943 | htra4 | 5 | 26329909 | 26350230 | protein\_coding |
| ENSDARG00000039272 | pls1 | 2 | 8262770 | 8313339 | protein\_coding |
| ENSDARG00000045306 | slc51a | 24 | 24816725 | 24837873 | protein\_coding |
